# Low hyper-oxygen exposure induces p21-dependent p53-independent senescence in alveolar cells

**DOI:** 10.7150/ijms.120133

**Published:** 2026-01-01

**Authors:** Cheng-Han Lee, Kan-Hsuan Lin, Ming-Sheng Lee, Chao-Jen Lin, Rei-Cheng Yang, Shih-Chung Wang, Chien-Sheng Hsu, Jun-Kai Kao

**Affiliations:** 1 Institute of Biomedical Sciences, National Chung Hsing University, Taichung, Taiwan.; 2 Frontier Molecular Medical Research Center in Children, Changhua Christian Children Hospital, Changhua, Taiwan.; 3 Department of Post-Baccalaureate Medicine, College of Medicine, National Chung Hsing University, Taiwan.; 4 Doctoral Program in Tissue Engineering and Regenerative Medicine, National Chung Hsing University, Taichung city, Taiwan.; 5 Department of Pediatrics, Kaohsiung Medical University Hospital, Kaohsiung City, Taiwan.; 6 School of Medicine, Kaohsiung Medical University, Kaohsiung City, Taiwan.

**Keywords:** bronchopulmonary dysplasia, alveolar epithelial cells, oxygen therapy, cellular senescence, autophagy, premature infants

## Abstract

Bronchopulmonary dysplasia (BPD) is a chronic pulmonary condition predominantly affecting premature neonates who necessitate oxygen therapy. Currently, BPD is classified into two types—old and new BPD—that differ in histology and pathology. The new BPD is observed in premature infants exposed to gentle ventilation and low oxygen concentrations, emphasizing the disruption of normal development. This study assessed the effects of low-to-high oxygen concentrations on rat alveolar epithelial L2 cells, aiming to mimic clinical scenarios. Exposure to 40 % oxygen induced p53-independent p21 expression in alveolar cells, resulting in G1-cell cycle exit cellular senescence. The inhibition of autophagy induced senolysis in L2 cells exposed to 40 % oxygen. Alveolar epithelial cells exhibit distinct responses to varying oxygen concentrations. Elucidating the interaction between senescence and autophagy is crucial for understanding the pathogenesis of novel bronchopulmonary dysplasia (BPD) in premature infants, thereby identifying potential preventive strategies.

## Introduction

Bronchopulmonary dysplasia (BPD) is a long-term lung condition that mainly impacts premature infants who need oxygen treatment. In cases of BPD, the airways sustain damage, resulting in the destruction of alveolar tissue. This condition predominantly affects infants born before 32 weeks of gestation with low birth weight, as their lungs are still in the canalicular or saccular stages of development. BPD is currently classified into two types—old and new. Old BPD was associated with aggressive mechanical ventilation in terms of peak pressure and hyperoxia (defined as a fraction of inspired oxygen > 60 %). New BPD affects premature infants with gentle ventilation and low oxygen concentrations. These two types differ in histology and pathology, with the new BPD characterized by the disruption of normal development 1, 2. Despite significant advances in neonatology, BPD remains a challenging condition of prematurity. Its multiple clinical phenotypes require further research to understand the complex multifactorial pathophysiology 3.

Oxygen therapy is crucial for the survival of critically ill premature infants. Nevertheless, it carries risks, as the presence of hydroxyl radicals (OH-) can induce oxidative stress in cells exposed to oxygen. Alveolar cells, located in the lung alveolus, are responsible for oxygen exchange. Immature alveolar cells in premature infants are specifically vulnerable to oxygen exposure owing to their underdeveloped antioxidant systems 4. Upon exposure to biochemical stress and DNA damage, alveolar cells initiate a range of defense mechanisms, including cell cycle arrest, senescence, autophagy, necrosis, and apoptosis. In the context of old bronchopulmonary dysplasia (BPD), ventilation employing high positive pressure and excessive volume may lead to alveolar cell death and significant local inflammation 5,6. However, numerous aspects of the pathogenesis, prevention, and treatment strategies of new BPD remain unclear.

Cellular senescence is a state in which cells cease to divide permanently due to persistent DNA damage and other stressors. These senescent cells are characterized by an increase in size, pH-dependent β-galactosidase activity 7, and alterations in gene expression 8,9. This condition leads to the formation of a bioactive secretome known as the senescence-associated secretory phenotype (SASP). Moreover, senescent cells contribute to the progression of several chronic diseases, such as diabetes, cancer, osteoarthritis, and Alzheimer's disease 10-12.

In this study, we hypothesized that exposure to low and high oxygen concentrations have different effects on alveolar cells. We conducted experiments using rat alveolar epithelial L2 cells, exposing them to varying oxygen levels to mimic clinical scenarios. Our results revealed that exposure to 40 % oxygen induced p53-independent p21 senescence in alveolar cells with interleukin (IL)-17 secretion.

## Material and Methods

### Cell culture

The rat alveolar epithelial L2 cell line (BCRC No.60276) was procured from the Bioresource Collection and Research Center in Hsinchu, Taiwan. These cells were cultured in Nutrient Mixture F-12 Ham medium, which was supplemented with 10% heat-inactivated fetal bovine serum, 1% streptomycin (100 μg/mL), and penicillin (100 U/mL). The culture conditions were maintained at a pH of 7.4 in a 5% CO_2_ incubator at 37 °C.

### Oxygen exposure of cells

L2 cells were seeded at a density of 1.2 × 10^5^ cells in 35 mm dish with 1.5 mL culture medium. After overnight, place the cells into the 5% CO_2_ incubator (SMA-165, ASTEC, Japan) with a preset oxygen concentration (40%, 60%, or 85%), and collect samples at the expected time points (24 hours, 48 hours, and 72 hours) for experimental analysis.

### Assessment of cell number (3-(4,5-dimethylthiazol-2-yl)-2,5-diphenyltetrazolium bromide [MTT] assay and cell counting)

Cell survival following oxygen exposure was assessed using MTT assay and cell counting.

### MTT assay

Cells were seeded at a density of 8 × 10^4^ cells per well in 100 μL of culture medium within tissue culture-grade 96-well flat-bottom microplates. The colorimetric assay employed is predicated on the reduction of the yellow tetrazolium salt (MTT) to purple formazan crystals by metabolically active cells, which are incubated for 4 hours at 37 °C and 5% CO_2_. The insoluble formazan crystals were subsequently dissolved in dimethyl sulfoxide, and the resulting solution was quantified by measuring the absorbance at 595 nm using a multi-well spectrophotometer.

### Cell counting

The number of L2 cells following oxygen exposure (24 hours, 48 hours, and 72 hours) was assessed using trypan blue staining and cell counting by LUNA-II™ Automated Cell Counter (Logos Biosystems).

### Cell proliferation

Cell proliferation following various treatments was evaluated utilizing a Click-iT^TM^ EdU (5-Ethynyl-2'-deoxyuridine) Cell Proliferation Kit (C10337; Thermo Fisher, US). L2 cells were seeded in 24-well plates and incubated overnight. After a 24-hour exposure to oxygen, the cells were treated with EdU in accordance with the manufacturer's protocol. EdU-positive cells were quantified using a fluorescence microscope (Olympus).

### Cell cycle analysis

The cells were harvested and subsequently washed with phosphate-buffered saline (PBS). The resulting pellet was fixed in cold 70% ethanol while vortexing to ensure thorough fixation and minimize clumping, followed by incubation for 30 minutes at 4 °C. The cells were then washed twice with PBS and centrifuged at 1000 x g for 5 minutes to prevent cell loss during the removal of the supernatant, particularly after ethanol removal. Subsequently, 200 µL of propidium iodide (PI) from a 50 µg/mL stock solution containing 5 µg RNase was added, and the mixture was incubated in the dark for 30 minutes at 37 °C. The DNA content of each sample was analyzed using a flow cytometer, with forward and side scatter measurements employed to identify single cells. PI exhibits a maximum emission at 605 nm, which is detectable using an appropriate bandpass filter.

### Detection of apoptosis

The FITC annexin V apoptosis detection kit with PI (640914, BioLegend) was employed to identify apoptotic and necrotic cells. Fluorochrome-labeled annexin V was utilized to specifically target and identify apoptotic cells. Since annexin V binding alone cannot differentiate between apoptotic and necrotic cells, a PI solution was employed. Early apoptotic cells excluded PI, whereas late-stage apoptotic and necrotic cells stained positively, due to the dyes entering the nucleus and binding to DNA. PI is a fluorescent dye that binds to DNA. When excited by 488 nm laser light, it can be detected in the PE/Texas Red® channel using a 610/10 bandpass filter. It is commonly used in flow cytometry to assess DNA content.

### SA-β-galactosidase staining

Senescence-associated-β-galactosidase (SA-β-gal) staining was conducted utilizing a Senescence β-Galactosidase Staining Kit (#9860; Cell Signaling, USA), adhering to the manufacturer's protocol with minor modifications. Following oxygen exposure, L2 cells were fixed with paraformaldehyde and subsequently stained for SA-β-gal. The stained cells were then photographed using a camera attached to a light microscope (Olympus Corporation, Tokyo, Japan) to evaluate the senescent cells in both the oxygen-exposed and control groups.

### Western blotting

L2 cells underwent the specified treatment regimen. The resulting cell pellet was lysed using radioimmunoprecipitation assay lysis buffer (#97063-270; VWR). The lysates were then centrifuged at 13,200 rpm for 15 minutes at 4 °C. Protein quantification was performed using the bicinchoninic acid assay (Thermo, US), following the manufacturer's guidelines. The proteins extracted were mixed with 5× loading buffer and heated. Equal amounts of protein were resolved on 8-12% sodium dodecyl sulfate-polyacrylamide gels and subsequently transferred to polyvinylidene fluoride membranes (ISEQ00010; Millipore, USA). The membranes were blocked with 5% skim milk in Tris-buffered saline with 0.01% Tween 20 (TBST) at room temperature for 1 hour. Following this, the membranes were incubated with primary antibodies against β-actin (1:10000, A1978; Sigma-Aldrich), p53 (1:1000, #2524; Cell Signaling), p-p53 (1:1000, AP0083; ABclonal), p21 (1:1000, A19094; ABclonal), LC3 (1:1000, L7543; Sigma-Aldrich), p62 (1:1000, #5114; Cell Signaling), pH2A.X (1:1000, #9718; Cell Signaling), p16 (1:1000, ARG42668, Arigo) and caspase 3 (1:1000, #9661; Cell Signaling). The membranes were washed three times with TBST for 10 minutes each and then incubated with a horseradish peroxidase-conjugated goat anti-rabbit antibody (1:10000, GTX213110-01, Genetex) or goat anti-mouse antibody (1:10000, GTX213111-01, Genetex). The target protein bands were visualized using the Fusion Fx system. β-Actin was employed as a loading control, and the band densities were quantified using ImageJ.

### Statistical analysis

The data are presented as the mean ± standard error. Statistical analysis was performed using the Prism software version 6.0 (Turku Center for Biotechnology University of Turku, Finland). The significance of the results was determined using ANOVA analysis of variance. Statistical significance was set at P < 0.05.

## Results

### Hyperxoia reduced L2 cell viability

**Figure 1:**
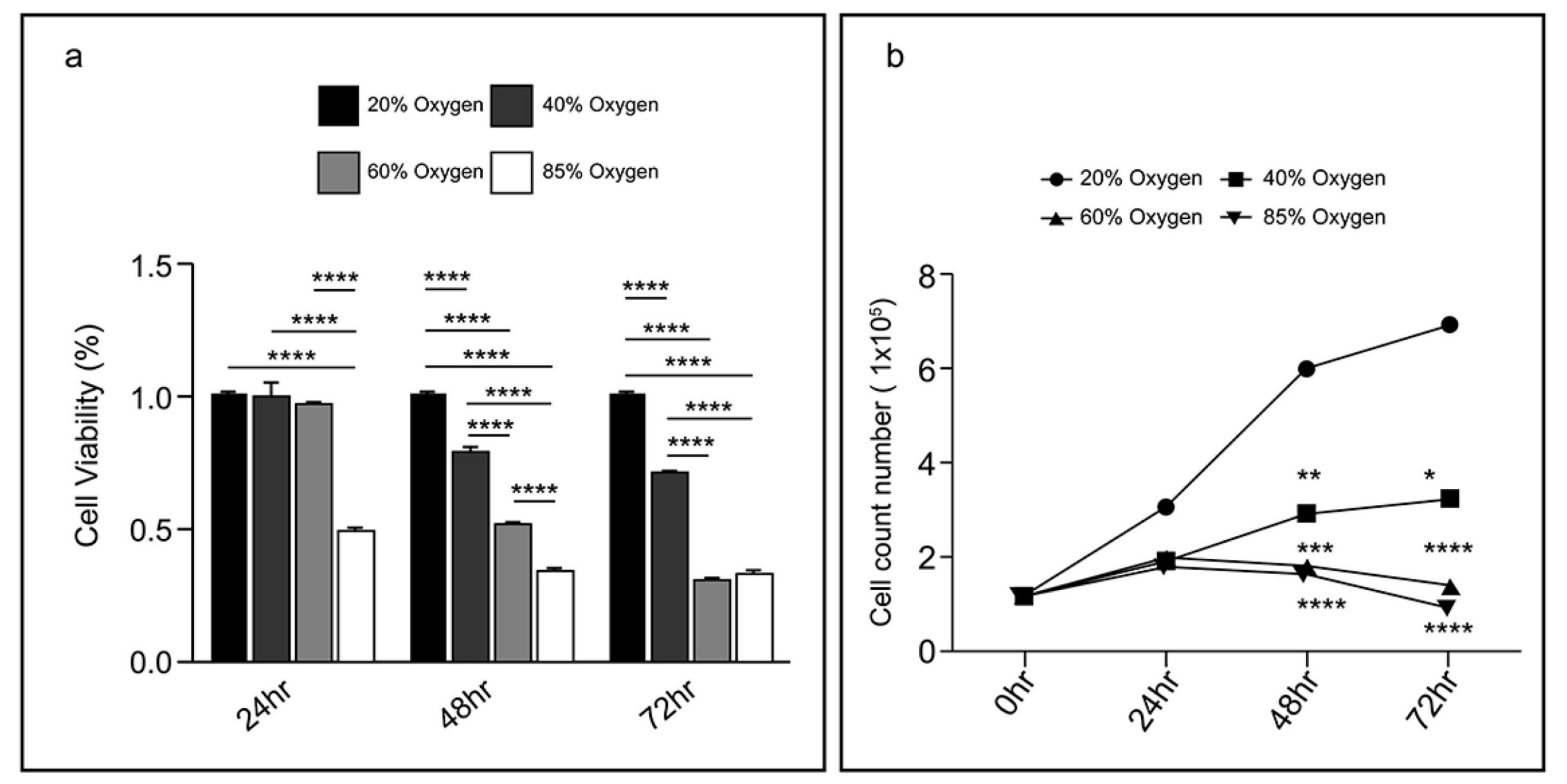
** Hyperoxia reduced L2 cellular viability: (a)** The results of bioactivity at varying oxygen concentrations and durations. Data are shown as mean ± SEM from three independent experiments. Statistical significance is indicated by *P < 0.05, **p < 0.01, ***p < 0.001 and ****P < 0.0001. **(b)** Live cell counting in various treatment groups. Data are shown as mean ± SEM. P-values was determined by One-way-ANOVA. Statistical significance is indicated by *P < 0.05, **P < 0.01, ***P < 0.001 and ****P < 0.0001, where values were compared to the 20% oxygen group by post hoc analysis.

After exposure to 40 % oxygen, L2 cell viability (measured by the MTT assay) was significantly reduced within the first 48 h and then remained at the same level (80 %). Exposure to 60 % oxygen caused a continuous decline in L2 cell viability from 24-72 h. Exposure to a high concentration of oxygen (85 %) reduced L2 cell viability to half at 24 h, and after 48 h, only 0.3 % viability remained. The bioactivity results at varying oxygen concentrations and durations are illustrated in Figure 1a. Additionally, trypan blue dye exclusion staining was used to measure the cell viability. Cells were counted manually using a hemocytometer, and the data revealed a reduction in cell number with increased oxygen exposure. After 48 h, the L2 cell count gradually reduced at oxygen concentrations above 60 %. However, the number of L2 cells exposed to 40 % oxygen is still slightly increased. Figure 1b illustrates the cell count results for different treatment groups. These results demonstrate that although 40 % oxygen exposure does not significantly reduce cell viability as much as high oxygen exposure, it inhibits cell proliferation.

### High concentration oxygen induced apoptosis in L2 cells

**Figure 2:**
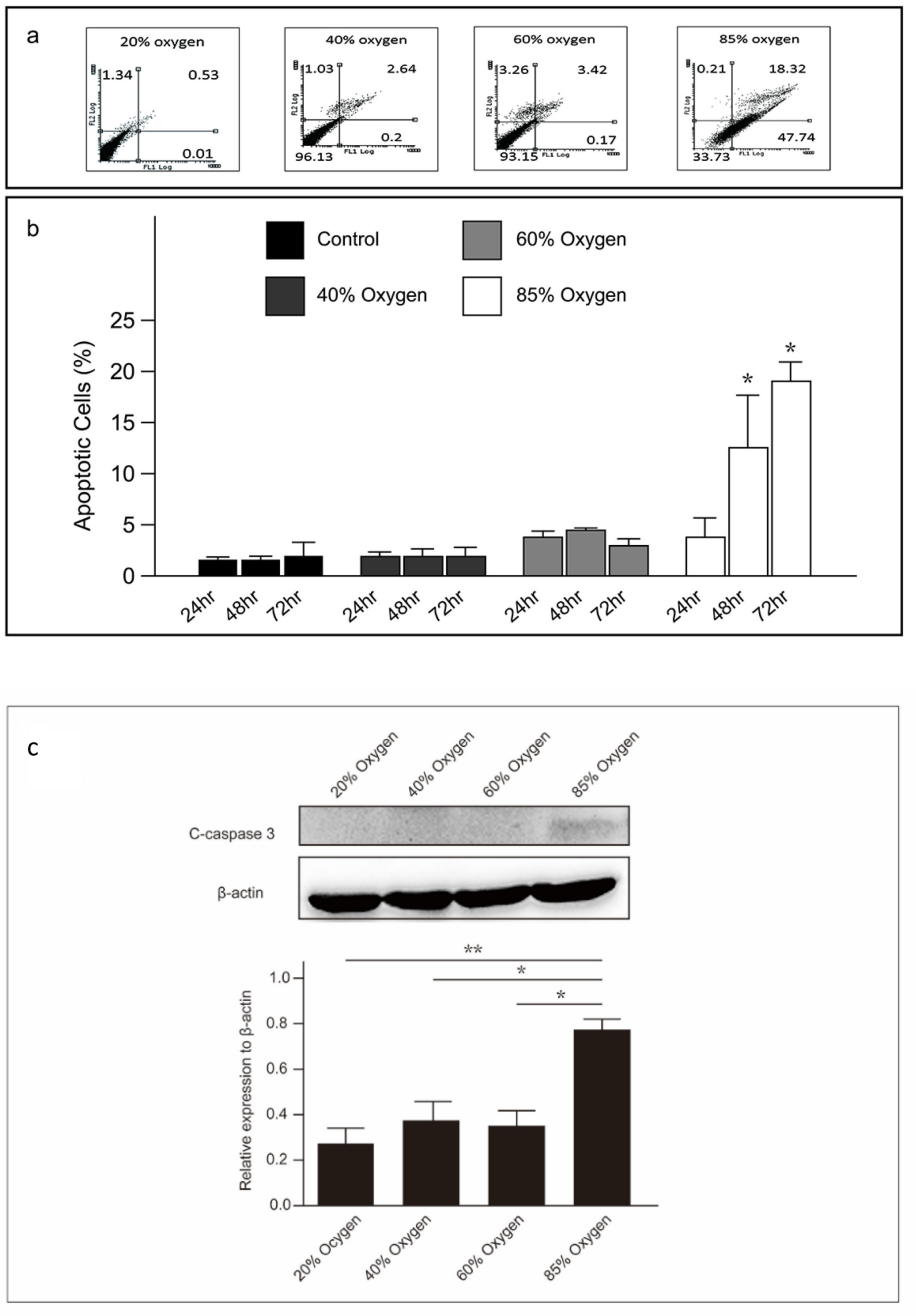
** High oxygen concentration induced apoptosis in L2 cells: (a)** The proportion of apoptotic L2 cells under varying oxygen concentrations, detected using annexin V and propidium iodide. **(b)** The percentage of apoptotic L2 cells at various oxygen concentrations and durations. **(c)** Representative western blot analysis shows the expression of cleaved-caspase-3 protein at various oxygen concentrations and durations. Quantification of cleaved-caspase3 abundance was normalized to the corresponding β-actin level. Data are shown as mean ± SEM from three independent experiments. P-values were determined by one-way ANOVA. Statistical significance is indicated by *P < 0.05, where values are compared to various treatment group at the same duration by post hoc analysis.

We assessed the apoptosis of L2 cells under varying oxygen concentrations using recombinant fluorescein-conjugated annexin V with PI. Live, non-apoptotic cells did not stain positively for either treatment. A flow cytometric analysis was performed after annexin V and PI staining of L2 cells under varying oxygen concentrations. Furthermore, Caspase-3, a pivotal executioner caspase involved in apoptosis, was quantified using immunoblotting techniques. The findings indicate that L2 cells underwent apoptosis when cultured at oxygen concentrations exceeding 60% after 48 hours (Figure 2a, 2b, and 2c).

### Varying oxygen concentrations induce different patterns of cell cycle arrest in L2 cell lines

**Figure 3:**
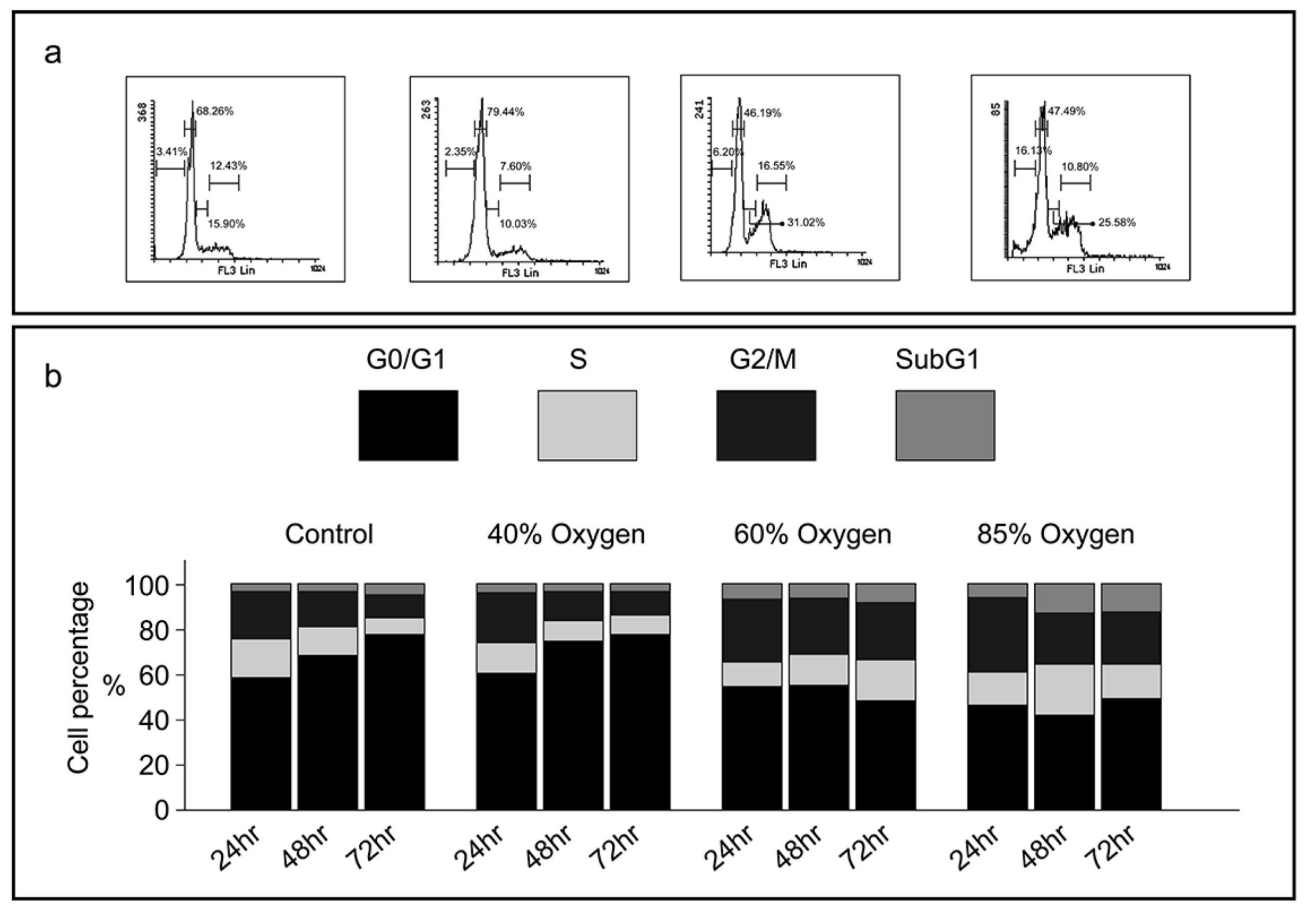
** Various oxygen concentrations induce different patterns of cell cycle arrest in L2 cell lines: (a)** Representative histograms demonstrating the proportion of L2 cells in each phase of the cell cycle under varying oxygen concentrations. **(b)** Mean percentage of cells in each phase of the cell cycle at various oxygen concentrations and durations. Cells are stained with propidium iodide. Means and standard error bars are based on four repeats. Cell cycle distribution is significantly blocked in the G0/G1 phase when L2 cells are cultivated in 40 % oxygen. At 85 % oxygen, the cell cycle is blocked in the G2/M phase, with an increase in apoptosis (subG1).

To elucidate the anti-proliferative mechanism of hyperoxia, we investigated its impact on cell cycle progression in L2 cells by employing oxygen concentrations of 40%, 60%, and 85% in cell culture. Flow cytometry analysis of the cell cycle demonstrated that after 48 hours, exposure to 40% oxygen significantly increased the proportion of cells in the G0/G1 phase (68-79%) while decreasing the percentage of cells in the S phase, thereby indicating a reduction in the proliferative cell population (Figure 3a and 3b). Conversely, exposure to 85% oxygen for 24 hours resulted in a significant accumulation of cells in the G2/M phase, and after 48 hours, there was a notable increase in the subG1 population. These results suggest that 40% oxygen effectively arrests the cell cycle at the G0/G1 phase, whereas 85% oxygen arrests the cell cycle at the G2/M phase and promotes apoptosis (subG1) (Figure 3).

### Forty percent oxygen induced senescence in L2 cells

**Figure 4:**
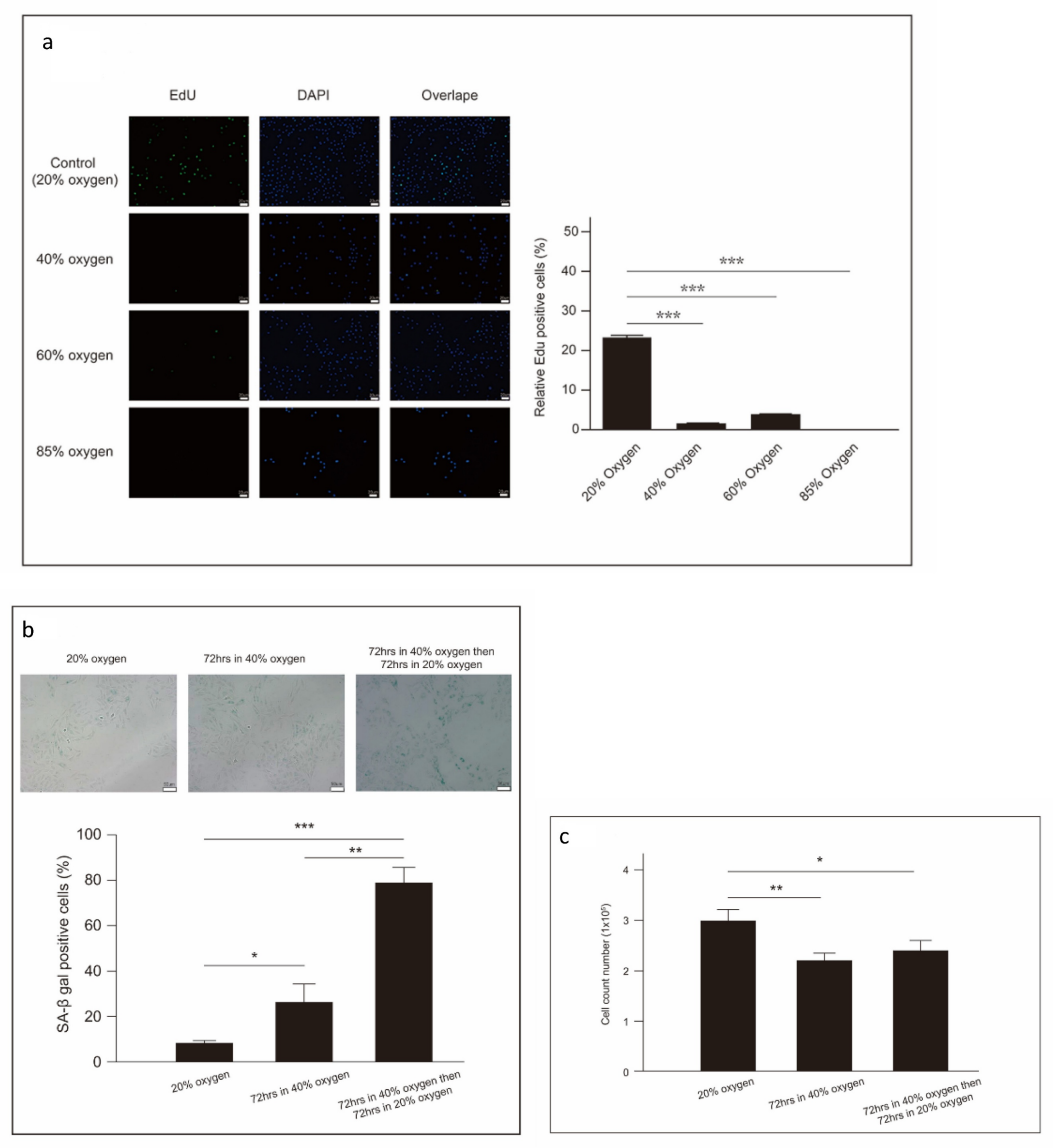
** Forty percent oxygen induced senescence in L2 cells: (a)** Cellular proliferation is measured using 5-ethynyl-2'-deoxyuridine immunochemical detection staining(green). Nuclei were stained with DAPI (blue), and the merged view shows the overlap. The quantification of EdU-positive cells was performed by counting cells from three random fields per group across three independent experiments. **(b)** Senescent cells of L2 cells are detected by β-galactosidase staining under 40 % oxygen exposure. Quantification of SA-β gal-positive cells was performed by counting cells from three random fields per group across three independent experiments. **(c)** Cellular senescence was stable and irreversible even return optimal growth conditions (20% oxygen environment). After oxygen exposure, collect the cells at the designated time points and count the total number of viable cells using a cell counter. Data are shown as mean ± SEM from three independent experiments. Statistical significance is indicated by *P < 0.05 and **p < 0.01.

To further evaluate the biological impact of 40% oxygen on L2 cell growth, cell proliferation was measured using the EdU assay, an immunochemical detection method that quantifies nucleotide analog incorporation into newly synthesized DNA. After 48 hours, the percentage of EdU-positive cells was significantly lower in the group exposed to 40% oxygen compared to the control group (Figure 4a). Additionally, β-galactosidase activity, a recognized marker of cellular senescence, was observed in L2 cells subjected to 40% oxygen. β-galactosidase staining at pH 6.0 was conducted on L2 cells cultured under both 40% and 20% (normal) oxygen conditions. Our results demonstrate that the proportion of SA-β-gal-positive cells in the 40 % oxygen-exposed group increased with increasing oxygen exposure time. To assess whether the effects of 40 % oxygen on L2 cells were reversible, we transferred cells to 20 % oxygen after 72 h of exposure to 40 % oxygen. The results, including both cell count and SA-β-gal-positive cell proportion, indicated that cellular senescence was stable and irreversible, even under optimal growth conditions 13 (Figure 4b and 4c).

### Forty percent oxygen induced senescence through p53-independent p21-dependent signaling pathway in L2 cells

**Figure 5:**
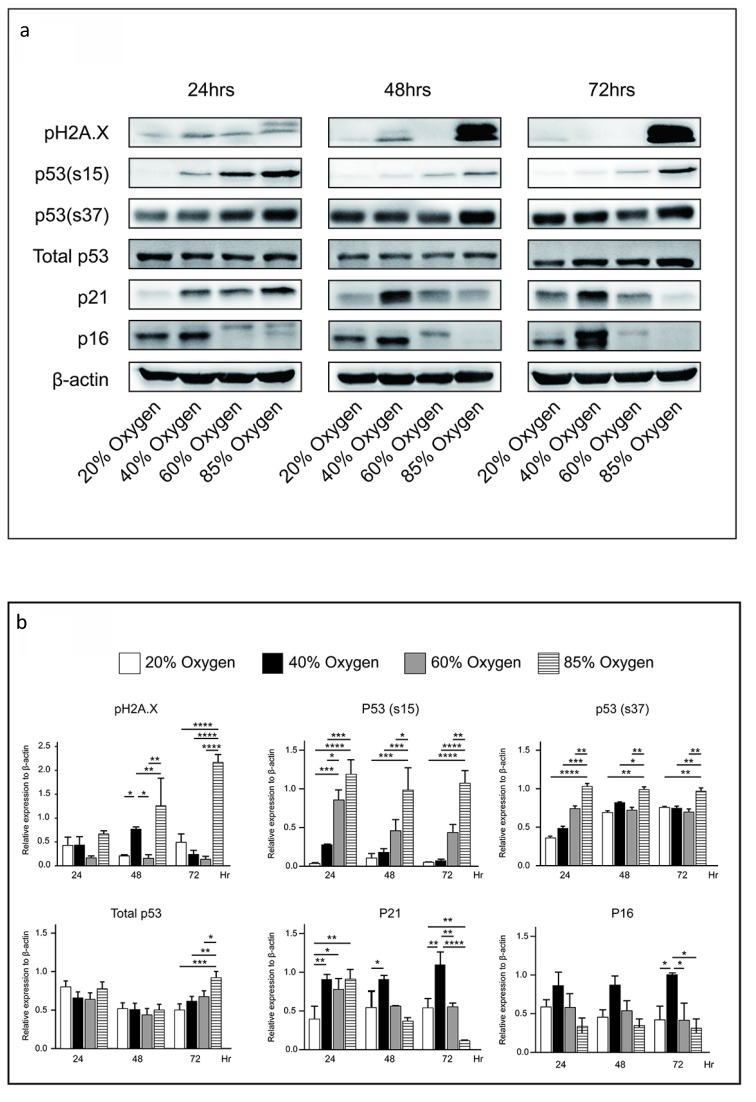

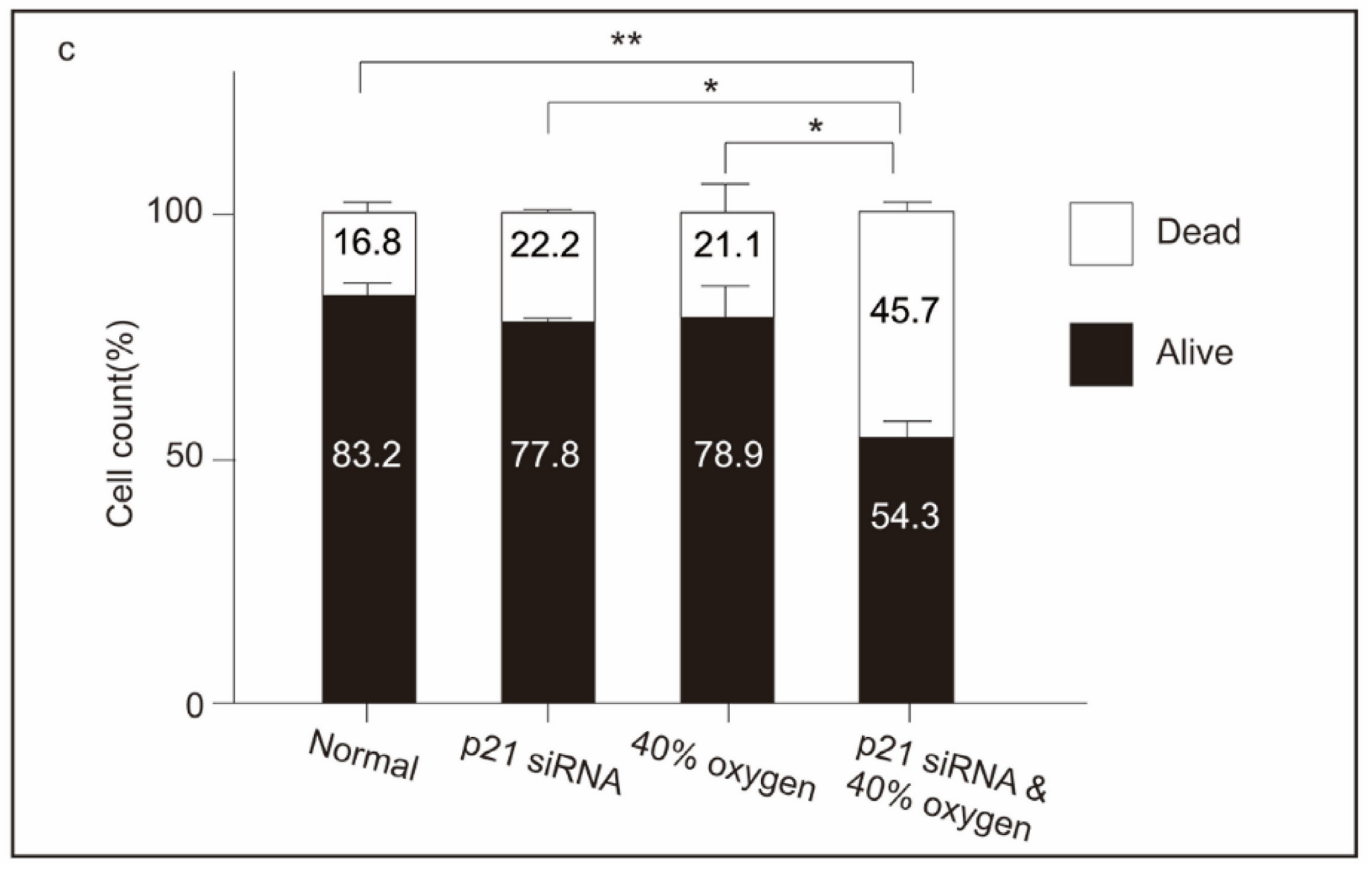
** Forty percent oxygen induced senescence through p53-independent p21-dependent signaling pathway in L2 cells: (a)** Representative western blot analysis of pH2A.X, p53, p53(s15), p53(s37), p16 and p21 protein expression at various oxygen concentrations and durations. **(b)** Quantification of the proteins shown in (A) was normalized to β-actin under corresponding oxygen concentrations and durations. **(c)** p21 small interfering RNA increased the death of L2 cells under 40 % oxygen exposure. Data are shown as mean ± SEM from three independent experiments. Statistical significance is indicated by *P < 0.05 and **p < 0.01.

While p53 is a primary transcriptional regulator of p21, and its mutation is linked to decreased p21 expression 14, the p53-independent induction of p21 has also been investigated by various research groups 15. We further explored the protein expression of p21 and p53 in L2 cells cultured under varying oxygen concentrations. Our results revealed that after a 48-hour culturing in 40 % oxygen, L2 cells significantly expressed p21 without increasing p53, phosphorylation of p53(s15), and phosphorylation of p53(s37) expression. Notably, phosphorylation of H2A.X (pH2A.X) was markedly increased after 48 hours of exposure to 40% oxygen. However, exposure to 85% oxygen resulted in even stronger pH2AX activation than 40% oxygen, suggesting more severe DNA damage. In contrast, L2 cells cultured in 85 % oxygen demonstrated increased p21 and p53 levels after 24 h. Subsequently, p53 production gradually increased, whereas p21 expression was reduced. The results demonstrated that 40% oxygen can enhance p21 production via a signaling pathway that does not depend on p53 activation (Figure 5a and 5b). Following this, we employed p21 small interfering RNA to knock down p21 and evaluate its impact on senescence triggered by exposure to 40% oxygen. The results demonstrate that p21 knockdown significantly increased L2 cell death under 40 % oxygen exposure (Figure 5c).

In examining the influence of species, we subjected human bronchial epithelium cells (BEAS-2B) and adenocarcinomic human alveolar basal epithelial cells (A549) to 40% oxygen for a duration of 72 hours. Similar to L2 cells, our findings indicated that both BEAS-2B and A549 cells exhibited cellular senescence and elevated p21 expression upon exposure to 40% oxygen. However, in contrast to the L2 cell line, an increase in the expression of total p53 and phosphorylated p53 was observed in BEAS-2B and A549 cells. Further experimentation utilizing A549 ^p53null^ cells revealed that exposure to 40% oxygen still compromised cell growth and induced senescence (Supplemental figure 2 and Supplemental figure 3).

### Senescent L2 cells secreted IL-17A after 40 % oxygen exposure

**Figure 6:**
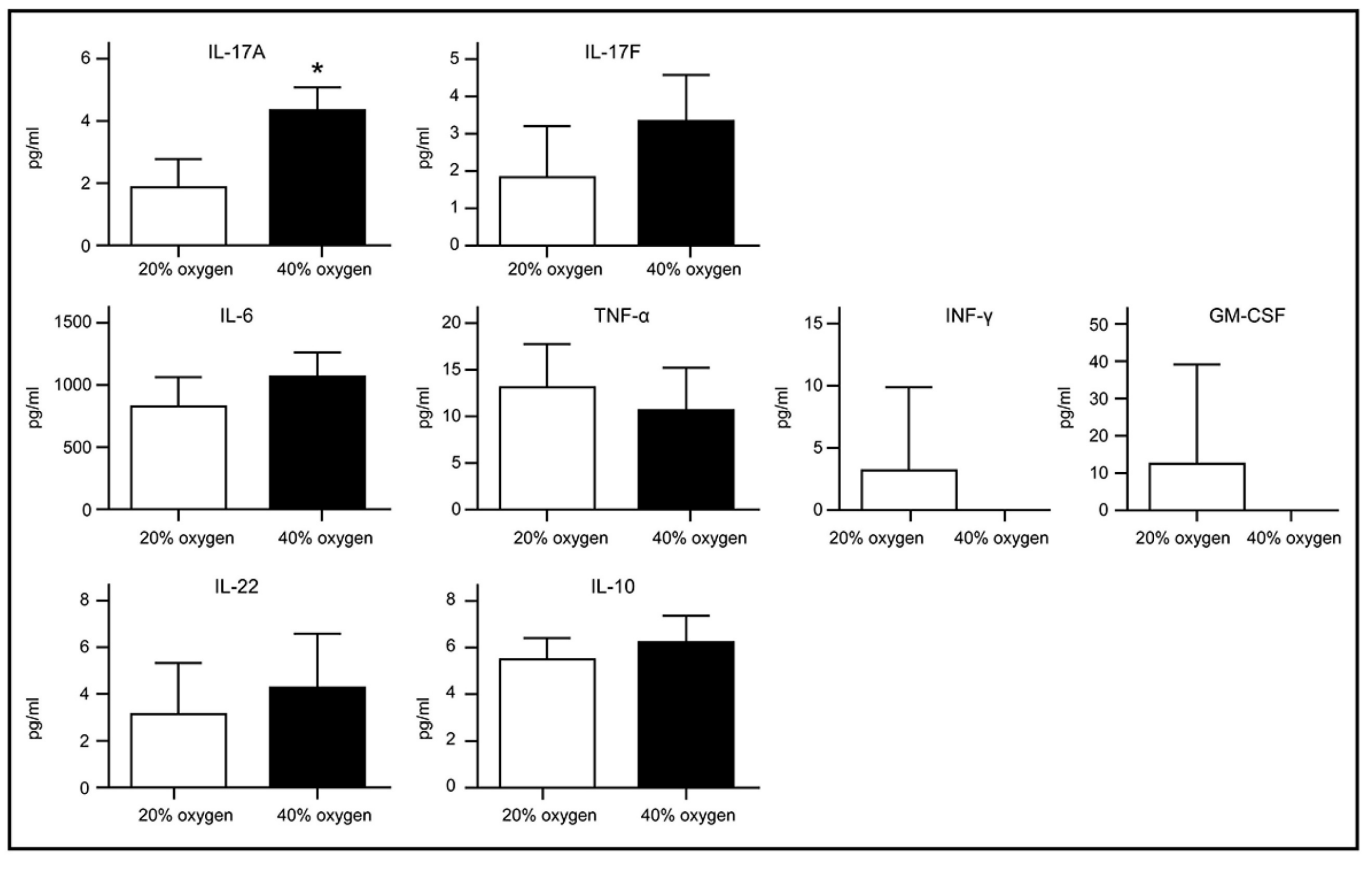
** Cytokines secretion by senescent L2 cells after exposure to 40 % oxygen are detected using enzyme-linked immunosorbent assay.** A significant increase in IL-17A compared to control group. Data are shown as mean ± SEM from three independent experiments. Statistical significance is indicated by *P < 0.05, **p < 0.01, ***p < 0.001 and ****P < 0.0001. Abbreviations: INF-γ, interferon-γ; TNF-α, tumor necrosis factor-α; GM-CSF, granulocyte-macrophage colony-stimulating factor.

Recent studies have shown that senescent cells release a variety of inflammatory cytokines, chemokines, growth factors, and matrix remodeling factors, which modify the local tissue environment and contribute to chronic inflammation and cancer. This phenomenon, known as the senescence-associated secretory phenotype (SASP), is observed in both cultured cells in vitro and in vivo 16, 17. We examined the levels of IL-17A, IL-17F, IL-8, IL-6, interferon-γ, tumor necrosis factor-α, granulocyte-macrophage colony-stimulating factor, IL-22, and IL-10 in the culture medium of L2 cells following exposure to 40% oxygen. Apart from a significant increase in IL-17A, no differences were noted in the other cytokines compared to the control group (Figure 6).

### Inhibition of autophagy induces cell death in senescent L2 cells

**Figure 7:**
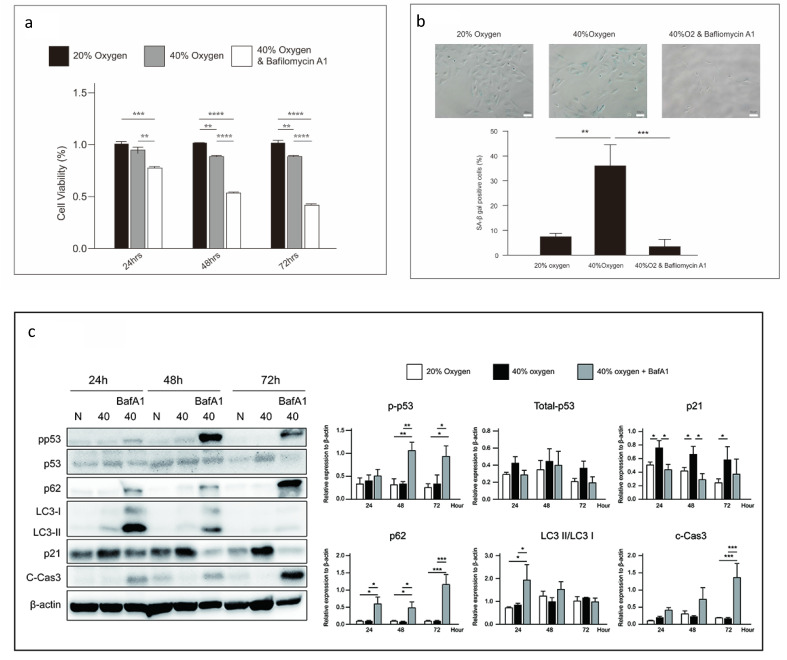
** Autophagy inhibition induces cell death in senescent L2 cells: (a)** Cellular viability is reduced by autophagy inhibitor, bafilomycin. Data are shown as mean ± SEM from three independent experiments. Statistical significance is indicated by *P < 0.05, **p < 0.01, ***p < 0.001 and ****P < 0.0001.** (b)** Bafilomycin reduces the levels of senescence-associated-β-galactosidase-positive cells in the 40 % oxygen group. Quantification of SA-β gal-positive cells was performed from three fields per group in three independent experiments.** (c)** Representative western blot analysis indicated protein expression at 40 % oxygen concentration and duration. Protein quantification was normalized to β-actin. Bafilomycin reduced p21 expression and increased p53 expression in L2 cells. Statistical significance is indicated by *P < 0.05, **p < 0.01 and ***p < 0.001.

Autophagy is a conserved cellular process, in which macromolecules undergo lysosomal degradation. It plays a crucial role in maintaining cellular quality control and energy homeostasis. Reactive oxygen species (ROS)-induced autophagy has been identified as a cellular protective mechanism that alleviates oxidative stress or is a destructive process 18, 19. In this study, autophagy was triggered in L2 cells after 40 % oxygen exposure. When autophagy was blocked by bafilomycin A1, the cell viability was significantly reduced (Figure 7a). However, autophagy inhibition reduced the proportion of SA-β-gal-positive cells (Figure 7b) and p21 expression and significantly increased p53 and caspase 3 expression (Figure 7c).

## Discussion

As reported by Hoffmann G, our results indicated that L2 cells serve as a suitable model for assessing human diseases associated with alveolar epithelial cells 20. In neonatal intensive care units, efforts are being made to maintain oxygen levels below 60 % to prevent hyperoxia-related damage. However, the potential harm caused by such oxygen concentrations to lung tissue remains unclear. This study demonstrated that prolonged exposure to 40 % oxygen can cause damage through mechanisms distinct from hyperoxia.

There is a growing recognition that infants with chronic lung disease following premature birth experience a clinical course and pathology distinct from those observed before the introduction of surfactants 21, 22. Unlike the old BPD, characterized by progressive stages with significant fibroproliferation, the new BPD often arises in preterm newborns who required minimal or no ventilatory support and were exposed to low oxygen levels during the early postnatal days 23. Autopsies reveal that the lung histology of these infants with the new BPD shows regions of more uniform and milder injury, yet impaired alveolar and vascular growth ^24.25,26^. Although certain reports have concluded that cellular senescence contributes to the progression of hyperoxic BPD 27, our findings demonstrated that senescent L2 cells without apoptosis play a crucial role when exposed to 40 % oxygen—aligning with the characteristics of the new BPD.

Apoptosis is vital for both the normal development of the lungs and their recovery following injury. When exposed to elevated oxygen levels, the imbalance between pathways that promote and inhibit apoptosis is key to understanding the causes of old BPD 28. In this study, exposure of L2 cells to high oxygen concentrations caused cell arrest in G2 phase owing to DNA damage, resulting in apoptosis. However, exposure to 40 % oxygen induced cell cycle exit in G1 phase (senescence), resulting in cellular senescence 29. Our findings showed that after 48 hours of exposure to 40% oxygen, L2 cells exhibited a significant increase in ROS, as measured by CM2-DCFDA (Supplemental Figure 1). We also observed an increase in the phosphorylated form of the H2A.X protein, a powerful indicator of DNA damage (Figure 5). These results reveal that as the accumulation of ROS and the severity of DNA damage increase, cells activate different response mechanisms.

Although senescent cells are in a state of arrest, they remain viable and actively contribute to various physiological processes, including embryogenesis, cellular reprogramming, tissue regeneration, wound healing, immunosurveillance, and tumor suppression 30,31,32. Senescent cells can facilitate or hinder disease development. The accumulation of senescent cells can result in the formation of atherosclerotic plaques. The loss of the replicative capacity of T cells and pancreatic β cells results in impaired tissue regeneration. The presence of senescent cells within critical physiological niches may disrupt tissue homeostasis 11. Notably, these cells demonstrate elevated metabolic activity 33 and actively inhibit apoptosis 34. Cellular senescence is characterized by sustained cell cycle arrest, transcriptional changes, the senescence-associated secretory phenotype (SASP), macromolecular damage, and dysregulated metabolism. In this study, senescent L2 cells exhibited increased secretion of IL-17A, a pro-inflammatory cytokine that is part of a family of six cytokines. IL-17A acts on barrier tissues, such as the lung epithelium, to promote inflammation.

It can recruit neutrophils to the mucosa and has been reported to play a role in various neonatal inflammatory disorders 35. Neonates at risk of developing BPD exhibited increased levels of IL-17A- and IL-17-associated lymphocytes 36,37. Blocking IL-17 signaling reduced lung inflammation and enhanced alveolarization in BPD animal models 38.

Although cellular senescence is generally considered irreversible proliferative arrest, various senotherapeutics are currently being developed, including senoreverters, SASP inhibitors, senolytics, and senomorphics 39,40. Senolytics, which induce the death of senescent cells, have been proposed as a means of enhancing human health. Autophagy and cellular senescence are two crucial cellular responses to various stressors. While autophagy was initially believed to inhibit cellular senescence by eliminating damaged macromolecules or organelles, it also promotes cellular senescence by facilitating the synthesis of senescence-associated secretory proteins 41-44. Our results demonstrated that autophagy inhibition facilitates apoptosis in senescent alveolar cells exposed to 40 % oxygen. Whether the autophagy induced in L2 cells in our study is anti-senescence or reflects a more complex interaction between autophagy and senescence requires further research.

DNA damage typically leads to the activation and stabilization of the p53 tumor suppressor protein, a critical event mediated by phosphorylation at various N-terminal residues, notably Serine 15 and Serine 37. When the damage is not yet severe, the activated p53 promotes the expression of downstream target genes such as p21, a cyclin-dependent kinase (CDK) inhibitor 45,46. p21 interacts with and inhibits the activity of cyclin E-CDK2 complexes, leading to the hypophosphorylation of retinoblastoma protein and subsequent cell cycle arrest at the G1-S transition 47. However, our findings suggest that the elevated levels of p21 observed in senescent L2 cells could independent of p53. Beyond p53, other transcription factors, including the Forkhead box O families, cAMP response element-binding protein/activating transcription factor, and OASIS, have been reported to bind to the promoter region of the p21 gene and regulate senescence 48,49,50.

The proteins p14, p16, and p21 are pivotal tumor suppressors that primarily function by regulating the cell cycle to prevent unchecked cell proliferation and the development of cancer. Specifically, p16 and p21 directly inhibit the CDK/cyclin complex, while p14 stabilizes p53 to induce cell cycle arrest. The p14 protein proved technically difficult to detect via antibody-based Western blotting in L2 cells. Therefore, we are unable to provide conclusive data regarding the role of p14 in the current study. Although there is substantial mechanistic overlap between the p16 and p21 signaling pathways in cellular senescence, our model demonstrates that p16 expression is not induced following L2 cells exposure to 40% oxygen. Moreover, p21 siRNA experiments confirm the operation of a p53-independent, p21-dependent pathway in this process (Figure 5a, 5b).

The observation that both BEAS-2B and A549 cells exhibit cellular senescence and increased levels of activated p53 and p21 upon exposure to 40% oxygen suggests a p53-mediated response. However, the finding that senescence and elevated p21 still occurred in A549 ^p53 null^ cells under the same hyperoxic (40% oxygen) conditions is highly significant. This collective data strongly suggests the existence of a p53-independent pathway that contributes to senescence and is primarily regulated by p21. These differential results across cell lines may be attributed to inherent biological differences in species (rat vs. human), tissue origin (alveolar vs. bronchial epithelium), and malignancy status (non-cancerous vs. cancerous). Further research is required to clarify the mechanisms underlying p21 induction and cellular senescence under 40 % oxygen exposure. A limitation of this study is that it was conducted in a cell model, and further verification through animal studies is required 51. Additionally, new BPD results from the interaction of multiple factors. In this study, we only analyzed the effect of oxygen exposure on alveolar development and therefore cannot fully explain the pathological mechanisms.

**Figure 8:**
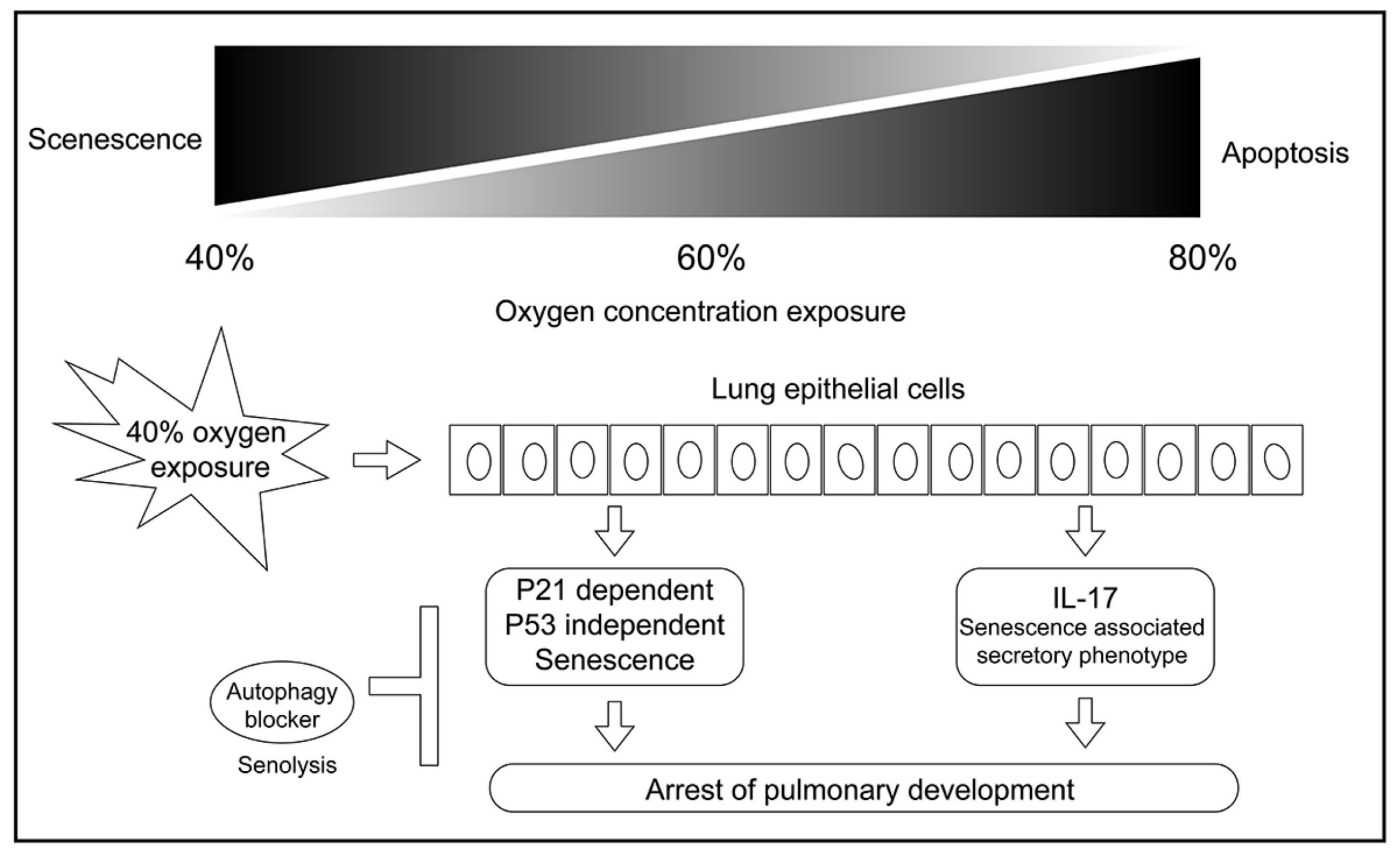
** The mechanism graph of this study.** Exposure to 40 % oxygen induced p53-independent p21 expression in alveolar cells, which caused G1 cell cycle exit and cellular senescence. Autophagy inhibition induced senolysis of senescent L2 cells.

In summary, our cell experiments confirmed that exposure to 40 % oxygen induced p53-independent p21 expression in alveolar cells, which resulted in G1 cell cycle exit and cellular senescence. Autophagy inhibition induced senolysis (Figure 8). Our results contribute to the pathogenesis of new BPD in premature infants and provide a potential preventive strategy.

## Supplementary Material

Supplementary figures.
