# Supplementary material for: Low hyper-oxygen exposure induces p21-dependent p53-independent senescence in alveolar cells
Source: Int J Med Sci. 2026 Jan 1;23(2):497–509. doi: 10.7150/ijms.120133 (PMC12825135; doi:10.7150/ijms.120133)
Supplement: Supplementary file 1 — Supplementary figures. [file ijmsv23p0497s1.pdf]

Supplemental figure 1

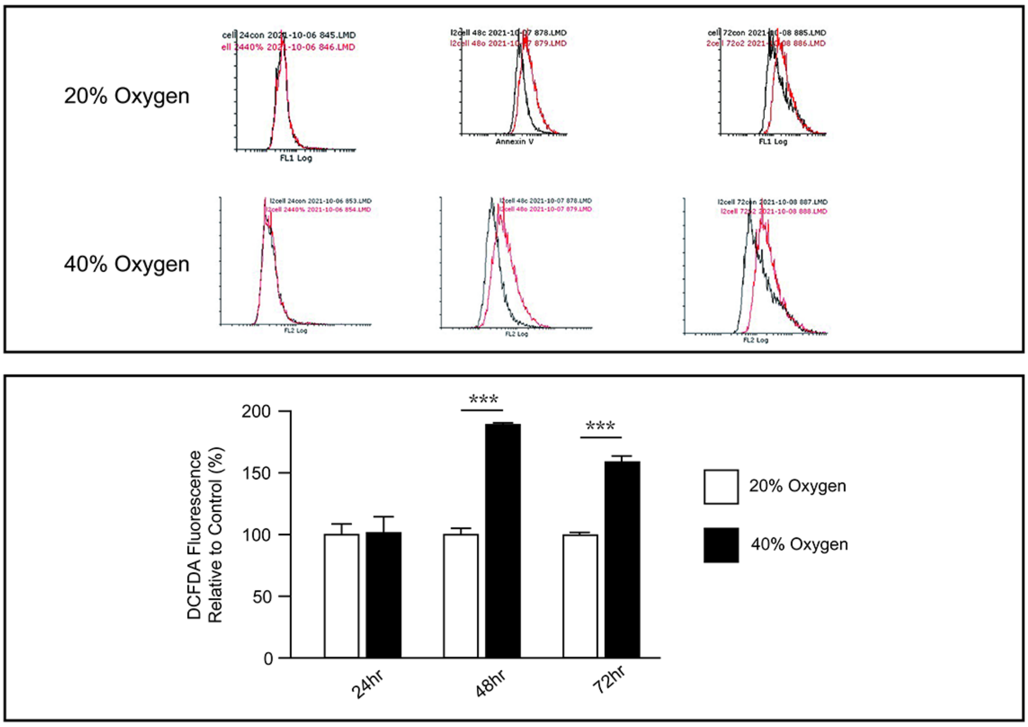

Supplemental Figure 1. Forty percent oxygen exposure induced oxidative stress in L2 cells. Intracellular ROS were detected using CM2-DCFDA fluorescence in L2 cells following 40% oxygen exposure for durations ranging from 24 to 72 hours. Data are shown as mean ± SEM from three independent experiments. Statistical significance is indicated by asterisks: $*P < 0.05$, $**p < 0.01$ and $***p < 0.001$.

Supplemental Figure 2

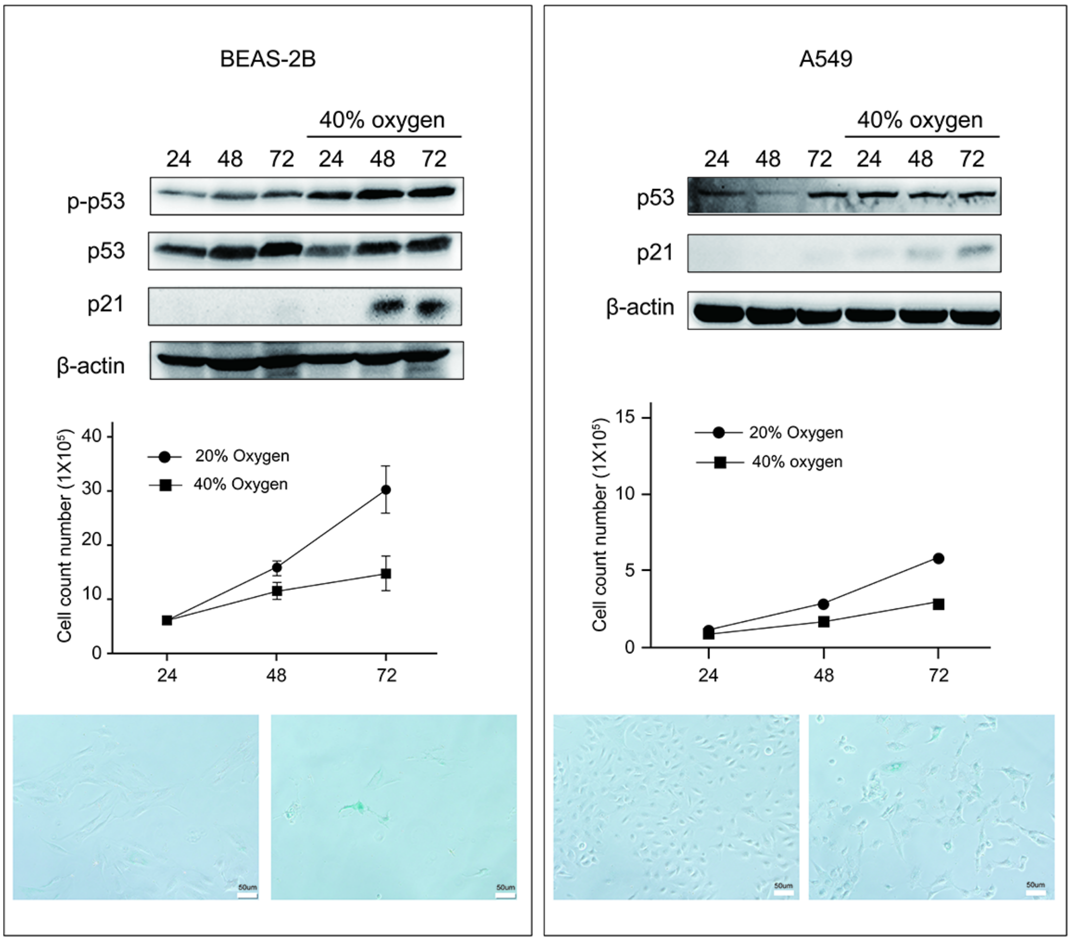

Supplemental Figure 2. Forty percent oxygen exposure induced senescence in BEAS-2B cells and A549 cells. The upper panel shows a representative western blot analysis indicated protein expression under 20% and 40% oxygen concentrations and various exposure durations. The Middle panel displayed cell counting data of BEAS-2B cells and A549 cells in various treatment groups. The lower panel illustrates senescent cells of BEAS-2B cells and A549 cells are detected by β-galactosidase staining under 40 % oxygen exposure.

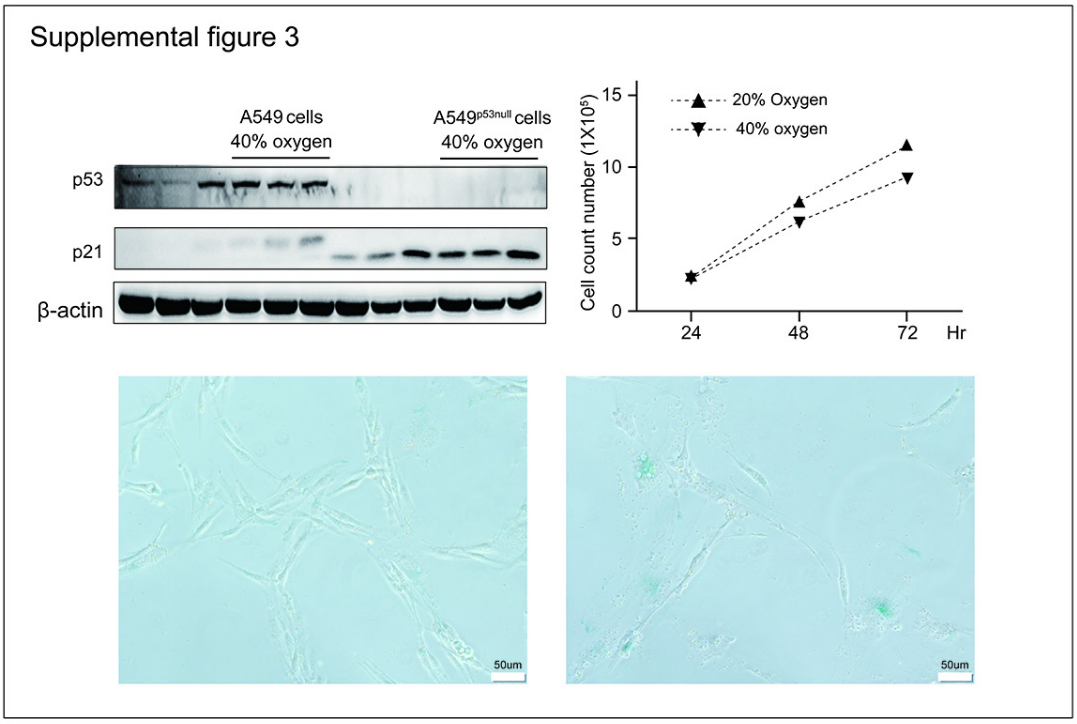

Supplemental Figure 3. **A549 p53 null cells exhibited senescence following forty percent oxygen exposure.** The upper-left panel displays a representative western blot analysis indicated protein expression in A549p53null cells under 20% and 40% oxygen concentrations and various exposure durations. The upper-right panel presents the corresponding cell counting data for A549p53null cells under the same 20% and 40% oxygen exposures and durations. The lower panel illustrates the detection of senescent A549p53null cells via β-galactosidase staining following exposure to both 20% and 40% oxygen.
